# Editorial: Industry and individuals: branding, labelling, and marketing of food products, volume II

**DOI:** 10.3389/fnut.2026.1881866

**Published:** 2026-06-11

**Authors:** Daniel Adrian Gârdan, Paweł Bryła, Ionel Dumitru, Iuliana Petronela Gârdan

**Affiliations:** 1 Department of Economic Sciences, Faculty of Economic Sciences, Spiru Haret University, Bucharest, Romania; 2 Doctoral School of Economic Sciences, “Dunărea de Jos” University of Galaṭi, Galaṭi, Romania; 3 Department of International Marketing and Retailing, Faculty of International and Political Studies, University of Lodz, Lodz, Poland; 4 Department of Marketing, Faculty of Marketing, Bucharest University of Economic Studies, Bucharest, Romania; 5 Department of Psychology and Education Sciences, Faculty of Psychology and Education Sciences, Spiru Haret University, Bucharest, Romania

**Keywords:** consumer behavior, dietary choices, food labeling, food marketing, food packaging, front-of-pack labeling, health symbols, nutritional literacy

The first volume of this Research Topic examined how industries—through branding, labeling, and marketing—shape consumer dietary choices, and how individuals respond to cues placed on the package, shelf, and screen (1). Volume II takes up the same line of inquiry, but at a moment when the food environment looks rather different. Rates of obesity and diet-related noncommunicable disease continue to climb across both high- and low-income settings (2); online food retail has scaled rapidly; and the front-of-pack labeling (FOPL) debate, once largely European, is now playing out in Latin America, Africa, and Asia under quite different regulatory conditions (3, 4). The aim of this second volume was to invite contributions that reflect this widened terrain, with attention to populations and methods underrepresented in the earlier collection.

Two trends in the recent literature framed our editorial work. The first is a growing recognition that food labels do not act on consumers in isolation: their effects are filtered through cultural identity, brand trust, health status, and the visual ecology of the package itself (5, 6). The second is the move from consumer-side studies toward research that also examines the supply side—how labeling shapes product reformulation, market entry, and competitive positioning (7). The 11 contributions here span experimental, survey-based, computational, audit-style, and conceptual work, addressing both currents. They were selected not to be comprehensive but to be cumulative: each one fills a corner of the picture the first volume left open.

Xu et al. offer what may be the volume's most provocative contribution. Across three experiments with 955 gastrointestinal disease patients, they show that implicit health symbols on probiotic packaging—microbiome-like icons, clinical typography, cool palettes—raise purchase intention, that the mechanism is health anxiety, and that the effect

strengthens under high disease threat. The finding sits uncomfortably with regulatory practice, which still focuses largely on text-based health claims (8). We read it as an argument for an effects-based approach to packaging oversight, one that asks what a design ensemble does to vulnerable consumers, not only what its individual elements assert.

Pérez-Armijo et al. arrive at a related conclusion from a different angle. In a quasi-experimental study of 5,140 Spanish adults with and without noncommunicable disease, they compared Nutri-Score and Warning Labels on five foods commonly thought of as healthy. The two schemes did not differ only in strength; they pointed in opposite directions. Nutri-Score reduced the share of participants rating these products as low quality, particularly for items graded A or B. Warning Labels did the opposite. The pattern held in both groups, which is the point: for adults with chronic disease, Nutri-Score appears to reinforce the very health halo it is meant to dispel, while Warning Labels do something closer to what FOPL was originally designed for.

Zhang moves the volume into less-charted theoretical territory. Three scenario-based experiments with 848 Chinese participants show that multi-dimensional identity labels—combining the economic-administrative identity of a Free Trade Zone with the cultural-territorial identity of a province—outperform single-identity labels for organic food. The effect runs through regional identity, moderated by regional attitudes. By bringing together Social Identity Theory and Multiple Social Categorization Theory, the paper opens a way of treating place as a layered construct rather than a single attribute—a useful corrective to the binary local-or-not framing common in the origin-labeling literature.

Tassy et al. offer something the broader literature has lacked: a supply-side, longitudinal view. Drawing on the Mintel Global New Product Database, they trace 327,194 newly launched packaged foods across 19 low- and middle-income countries between 2015 and 2023. The composition of the supply has shifted—fewer cookies, cakes, and still drinks; more meat, poultry, coffee—and median sugar has fallen while protein has risen. Where mandatory FOPL schemes have been introduced, further reductions in sugar and, often, sodium follow; voluntary schemes show weaker effects. The study reframes labeling as a lever for reformulation, not only for consumer information.

Three further papers ask who labels and interventions actually reach. Dai and Wu work at the scale online catering allows-−44,050 Chinese consumers—comparing three families of nudge-based interventions. Nudges work, but unevenly: women, lower-income consumers, those with less formal education, and patients with chronic disease respond more strongly than other segments, and decision-structure interventions outperform decision-aid ones among economically constrained groups. Nyarko and Bartelmeß stay with segmentation but shift to West Africa, using MaxDiff and five supervised machine-learning models to identify what 200 urban Ghanaian consumers weigh when choosing multinational products; the gender contrast is sharp. García-Salirrosas et al. provide the volume's most explicit test of the literacy-to-behavior link, using PLS-SEM on 637 Peruvian consumers to show that nutritional literacy influences willingness to consume healthy brand foods both directly and through healthy lifestyle behavior.

Two papers turn the lens on the scaffolding around labels. Iheme et al. report what is, to our knowledge, the first systematic audit of pre-packaged food labels against Nigeria's NAFDAC requirements-−883 products across nine state capitals. Overall compliance is high, but the gaps are revealing: quantitative ingredient declaration sits at 39%, with locally manufactured products lagging behind imports. Liu and Chen surveyed 2,517 Chinese college students and reached a finding we did not expect: having taken a formal nutrition course was negatively associated with awareness of nutrition labels. Frequency of use, demand for information, and trust in labels were the real predictors, with online video the dominant channel.

Two contributions occupy more particular niches but earn their place. Reynolds and Soedamah-Muthu propose, in the volume's only opinion piece, a clearer terminology and a structured order for declaring financial support in nutrition communication—distinguishing disclosure from acknowledgment in a way the field has long needed (9). Mouatadid et al. take readers to a market the literature rarely visits—aged cheese in Morocco—documenting an emerging category in which local origin and income shape purchase, with a clear strategic opening for domestic producers.

Read together, these 11 contributions in the Research Topic make a cumulative argument. The persuasive influence of packaging has migrated, in part, away from explicit propositional content toward implicit symbolic cues that current regulation barely sees. The same label can produce divergent effects depending on consumers' chronic disease status, gender, regional attachment, or trust in the label itself. The geography of the conversation has expanded, and with it the empirical base. Running through everything is the question of credibility—of labels, of the science that evaluates them, and of the disclosures that surround both. We hope readers find here both a snapshot of the field as it stands and an agenda for what remains to be done.
